# Laparoscopic Liver Resection Utilizing a Water Jet Scalpel for Patients With Liver Fibrosis

**DOI:** 10.7759/cureus.45212

**Published:** 2023-09-14

**Authors:** Yusuke Uemoto, Takahisa Fujikawa, Taisuke Matsuoka

**Affiliations:** 1 Surgery, Kokura Memorial Hospital, Kitakyushu, JPN

**Keywords:** hepatocellular carcinoma, liver cirrhosis, liver fibrosis, water-jet scalpel, laparoscopic liver resection

## Abstract

Introduction

A variety of devices are utilized in order to resect liver parenchyma in laparoscopic liver resection. However, liver fibrosis makes hepatectomy problematic because the liver is rigid and prone to bleeding. The water jet scalpel, which dissociates the liver parenchyma with a jet stream has no thermal damage and is clinically utilized in liver resection, but its safety and efficacy during laparoscopic liver resection for patients with liver fibrosis remain unknown.

Methods

We analyzed patients who underwent laparoscopic liver resection utilizing the water jet scalpel with liver fibrosis at our hospital. A water jet scalpel was used for liver parenchymal transection, and a saline-linked ball-tipped electrocautery was simultaneously used for hemostasis.

Results

Subsectionectomy was one case, left lateral sectionectomy was two cases, and non-anatomical liver resection was three cases. The median blood loss was 70 mL (24-104 mL). There was no need for the intraoperative Pringle's maneuver. No perioperative blood transfusion was performed, and there were no postoperative complications, including posthepatectomy liver failure.

Conclusion

It was suggested that laparoscopic liver resection in patients with liver fibrosis can be safely performed with the water jet scalpel.

## Introduction

Laparoscopic liver resection (LLR) for hepatocellular carcinoma (HCC) and metastatic liver tumors has become widespread with the improvement of surgical instruments and procedures [1]. LLR has smaller scars and is associated with less blood loss, a lower postoperative complication rate, and a shorter length of stay compared to open liver resection [2].

Various methods or devices were utilized for parenchymal transection in LLR, including the clamp crushing technique [3], the Cavitron ultrasonic surgical aspirator (CUSA) [4], and the water jet scalpel (WJS) [5]. WJS dissociates the liver parenchyma with the jet stream, and fine vessels can be selectively preserved without thermal damage [6]. In actuality, WJS has been reported to reduce blood loss and liver resection time [5,7].

Hepatectomy is a successful radical treatment for HCC, and it is occasionally required in patients with severe liver fibrosis. In liver fibrosis, it is difficult to crush or dissociate the liver parenchyma while preserving fine blood vessels [8,9]. Additionally, a fibrotic liver usually has portal hypertension, which makes even small vessel injuries result in spurt hemorrhage. Even with WJS, liver resection is still considered difficult and frequently bleeds.

In a previous report, we described secure and effective hemostasis using a combination of saline-linked, ball-tipped electro-cautery (SLiC), and wet oxidized cellulose [10,11]. In this procedure, continuous superficial low-temperature heat coagulation by the use of SLiC is crucial for prompt hemostasis. Since WJS employs saline, it can be readily combined with SLiC, and by using both together, we think that reliable parenchymal transection could be achieved even in patients with liver fibrosis.

In the current report, we demonstrate LLR using the WJS and SLiC combination technique in patients with liver fibrosis and describe the case results.

## Materials and methods

We investigated patients who underwent LLR for HCC using WJS from 2021/08 to 2021/10 at our institution. Clinical data collected from electronic medical records included sex, age, etiology, Child-Pugh (C-P) score, and indocyanine green retention rate at 15 min (ICGR-15). Computed tomography (CT) or magnetic resonance imaging (MRI) was performed on all patients to identify the tumor. Operation time, intraoperative blood loss, performing blood transfusions and Pringle's maneuver (PM), postoperative length of stay, and perioperative complications were investigated as perioperative outcomes.

Oncological factors were evaluated pathologically in accordance with the Japan Liver Cancer Study Group [12]. The stage of pathological liver fibrosis was determined using the METAVIR score [13]. Finally, we focused on patients with pathologically proven liver fibrosis. The Kokura Memorial Hospital Clinical Research Ethics Committee authorized the protocol of the current study (#21021002), which complied with the Declaration of Helsinki.

Surgical technique

LLR was generally performed according to the method described previously [14], which was modified for the use of WJS. Briefly, the primary surgeon dissected the liver parenchyma with laparoscopic coagulating shears (LCS; Ethicon, Cincinnati, Ohio) and/or WJS (ERBEJET2; ERBE Elektromedizin, Tübingen, Germany) while the secondary surgeon controlled bleeding with a SLiC plugged into an electrosurgical VIO device (ERBE Elektromedizin GmbH, Tübingen, Germany).

The resection line was pre-coagulated with SLiC, and the liver surface was first cut with LCS. Subsequently, liver parenchymal transection was performed with WJS. Especially when hepatic fibrosis is severe, the WJS tip could be firmly against the tissue to prevent the splash and progressively moisten and soften the tissue. SLiC was used to control the bleeding when it occurred. If stopping the bleeding was difficult, the SLiC combined with wet oxidized cellulose (SLiC-WOC) method was performed [10,11]. Large vessel dissections can also be carried out this way. The WJS tip was pressed against both sides of the vessel and slowly moved along the longitudinal axis. Water flow turned around to the dorsal side of the vessel as the vessel dissection progressed, and the dorsal side was gradually dissected. Additionally, water flow might be confirmed from the contralateral side (like a "fountain sign" or "liquefaction sign"). Finally, the dissected vessel was clipped and cut.

The procedures of laparoscopic left lateral sectionectomy using WJS are shown in Video 1. Liver parenchymal transection was started from the caudal side. WJS was used to conduct adequate dissection around the ventral and dorsal sides of the Glissonean branch of segment 3 (S3). The WJS was slowly moved in parallel with the vessel to perform the dissection. The dissection of the Glissonean branch by WJS was able to proceed slowly and surely because there was no fear of thermal damage to the Glissonean branch. The Glissonean branch of S3 was clipped and cut. The Glissonean branch of segment 2 and the left hepatic vein were similarly dissected using WJS and cut.

**Video 1:** Laparoscopic left lateral sectionectomy utilizing the water jet scalpel in a patient with liver cirrhosis caused by hepatitis B virus infection

The procedures of laparoscopic non-anatomical liver resection of segment 8 (S8) using WJS are shown in Video 2. The tip of the WJS was pressed firmly against the liver tissue to avoid splashing and was moved slowly along the liver resection line. Hard liver tissue was gradually moistened. Bleeding occurred sometimes due to portal hypertension, but bleeding was stopped appropriately with SLiC. Perivascular dissection was made easier by sufficiently softening the liver tissue. A peripheral Glissonean branch of S8 was clipped and cut.

**Video 2:** Laparoscopic non-anatomical liver resection utilizing the water jet scalpel in a patient with liver cirrhosis from chronic hepatitis C

## Results

The background characteristics of six patients with liver fibrosis who underwent LLR using the WJS are shown in Table 1. There was one case of subsectionectomy, two cases of left lateral sectionectomy, and three cases of non-anatomical resection. Primary diseases included two cases of hepatitis C virus infection, three of hepatitis B virus infection, and one of nonalcoholic steatohepatitis. Most cases were classified as Child-Pugh class A, but pathological investigation revealed all six cases were categorized as fibrosis stage 2 or higher.

**Table 1:** Background characteristics of patients in the current case series C-P; Child-Pugh, ICGR-15; indocyanine green retention rate at 15 min, F; female, M; male, NASH; nonalcoholic steatohepatitis, HBV; hepatitis B virus, HCV; hepatitis C virus

Case No.	Age	Sex	Primary disease	Location	Operative procedures	C-P classification	ICGR-15 (%)	Fibrosis stage
1	81	F	NASH	S3	Subsectionectomy	A	16.2	4
2	77	F	HBV	S4/5	Non-anatomical resection	B	40.8	4
3	72	F	HBV	S2/3, S6	Left lateral sectionectomy, non-anatomical resection	A	24.5	4
4	80	M	HCV	S8	Non-anatomical resection	A	12.5	2
5	75	M	HCV	S5, S8	Non-anatomical resection	A	13.1	2
6	75	F	HBV	S2	Left lateral sectionectomy	A	6.9	3

**Figure 1:**
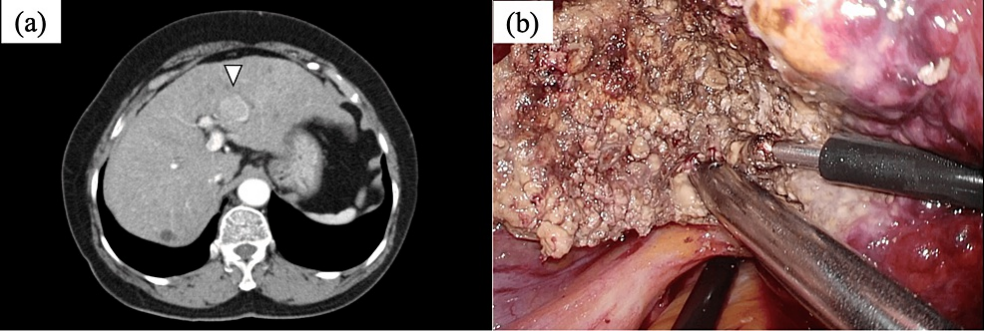
CT images and intraoperative findings of case 1 (a) A 3-cm-sized tumor in the S3 section of the liver was detected. (b) WJS was used to conduct laparoscopic S3 subsectionectomy. CT; computed tomography, WJS; water jet scalpel, S3; segment 3

**Figure 2:**
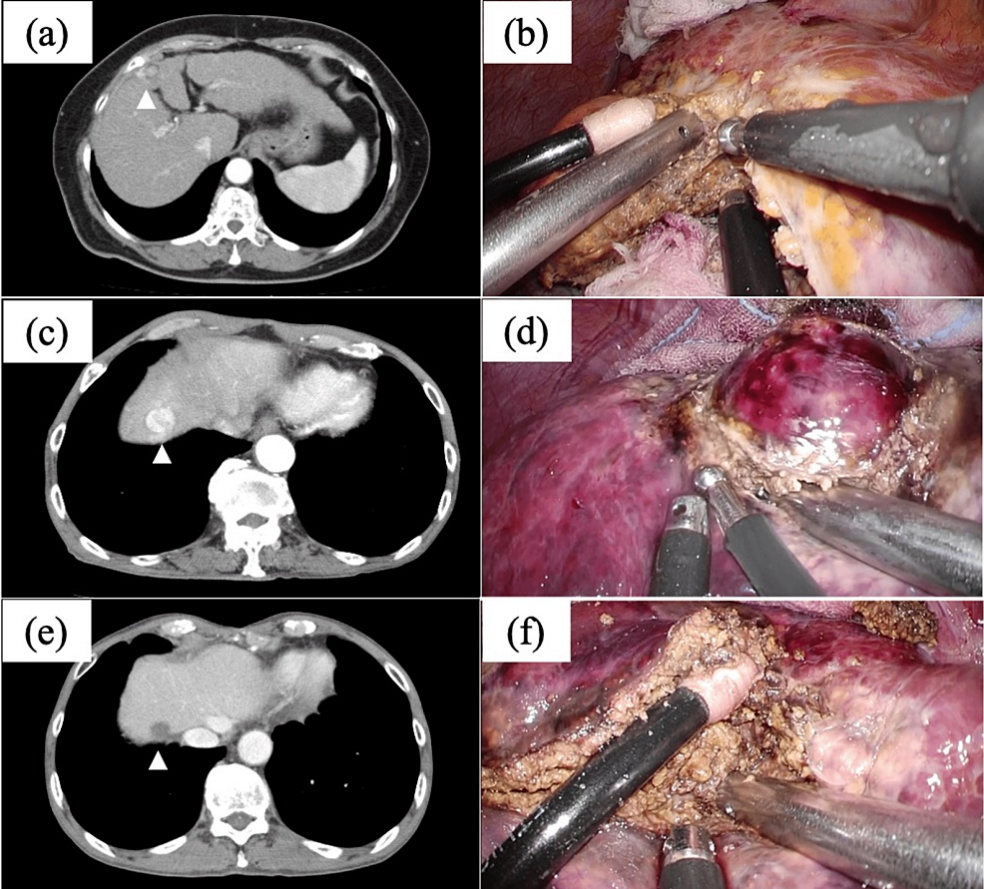
CT images and intraoperative findings of cases 2, 4, and 5 (a, b) In case 2, a 2.5-cm-sized tumor in S4/S5 was detected (arrowhead), and non-anatomical liver resection was performed utilizing WJS. (c, d) In case 4, a 1.5-cm-sized tumor in S8 was well-enhanced in an arterial phase (arrowhead), and S8 non-anatomical liver resection was performed using WJS. (e, f) In case 5, a well-demarcated 2.0-cm-sized tumor was detected in S5/S8, and non-anatomical liver resection of S5/S8 was performed in the same manner. CT; computed tomography, WJS; water jet scalpel, S4; segment 4, S5; segment 5, S8; segment 8

Case 1 had liver cirrhosis caused by nonalcoholic steatohepatitis. A 3-cm-sized HCC in segment 3 (S3) was detected, and laparoscopic S3 subsectionectomy was performed (Figure 1). Cases 2, 4, and 5 had liver cirrhosis caused by hepatitis virus infection, and the tumors were detected in segment 4/5, segment 8, and segment 5/8, respectively. Laparoscopic non-anatomical liver resection was performed in each case (Figure 2).

**Figure 3:**
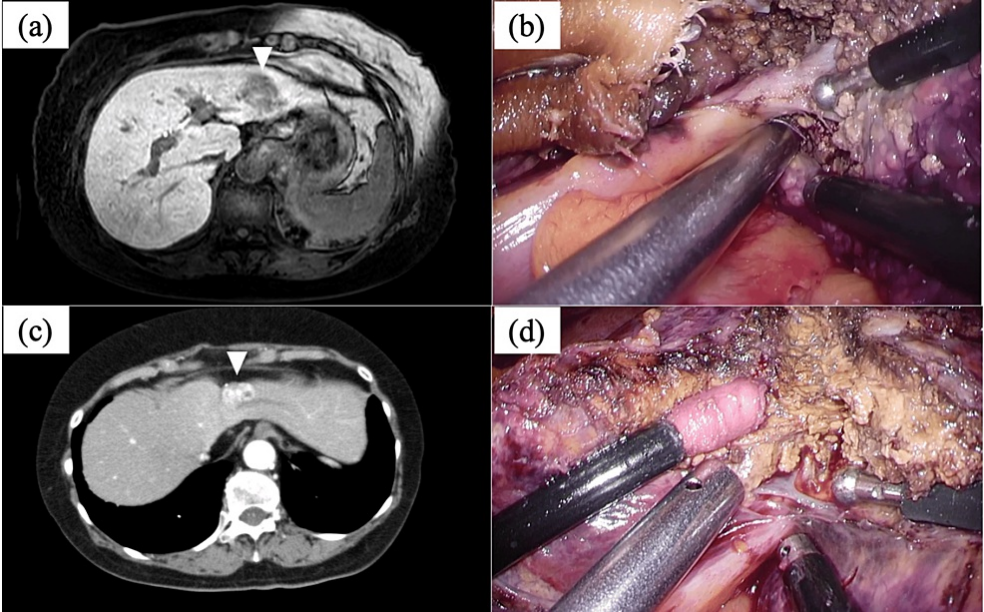
MRI image and intraoperative findings of cases 3 and 6 (a, b) In case 3, a 3-cm-sized tumor in the lateral section of the liver was detected in contrast-enhanced MRI (arrowhead), and left lateral sectionectomy utilizing WJS was performed. (c, d) In case 6, a 2.5-cm-sized tumor was detected in S2 and the patient underwent left lateral sectionectomy using WJS. MRI; magnetic resonance imaging, WJS; water jet scalpel, S2; segment 2

Case 3 had liver cirrhosis caused by hepatitis B virus infection. A 3-cm-sized HCC in the lateral section of the liver and a 1.5-cm-sized HCC in segment 6 of the liver were discovered, and a laparoscopic left lateral sectionectomy and non-anatomical hepatectomy of segment 6 were performed (Figures 3a, 3b). Case 6 had liver cirrhosis caused by hepatitis B virus infection, and laparoscopic left lateral sectionectomy for a 3-cm-sized HCC in the lateral section was undertaken (Figures 3c, 3d).

The perioperative outcomes of patients in the current case series are shown in Table 2. The median operative time and blood loss were 301 (162-580) min and 70 (24-104) mL, respectively. Taping of the hepatoduodenal ligament was performed in all cases and PM was planned as necessary, but none required it. No perioperative blood transfusions were necessary. The postoperative course was uneventful without complications, including posthepatectomy liver failure (PHLF), and the median length of postoperative stay was seven (6-8) days. Pathological findings revealed that the surgical margin was negative in all six cases.

**Table 2:** Perioperative outcomes of patients in the current case series Op-T; operation time, BL; blood loss, B-T; blood transfusion, PM; Pringle's maneuver, LOS; length of stay

Case No.	Operative procedures	Op-T (min)	BL (mL)	B-T	PM	LOS (day)	Surgical margin	Stage
1	S3 subsectionectomy	382	94	None	None	6	Negative	I
2	Non-anatomical resection	162	34	None	None	7	Negative	II
3	Left lateral sectionectomy, non-anatomical resection	219	75	None	None	8	Negative	IV
4	Non-anatomical resection	183	24	None	None	6	Negative	I
5	Non-anatomical resection	580	65	None	None	7	Negative	III
6	Left lateral sectionectomy	395	104	None	None	7	Negative	II

## Discussion

We explained how WJS is used during LLR in patients with liver fibrosis. WJS using saline is conveniently used in combination with SLiC, implying that it helps with bleeding control. In addition, dissection of the Glissonean vessels can be done securely, even though it takes time to moisten and soften the cirrhotic liver with WJS. Accordingly, it may be concluded that WJS is beneficial for laparoscopic liver resection even in patients with liver fibrosis.

It is reported that WJS demonstrates a high level of tissue selectivity [5,7]. That is, it is possible to proceed with liver resection while performing proper vascular dissection. When the jet stream of WJS is applied to the edge of the vessel, the dorsal side of the vessel is dissected. This is because the Coanda effect causes the water flow to wrap around the dorsal side of the vessel. The utility of WJS in liver resection has been reported in previous studies. It has been utilized in open and laparoscopic liver resection [5], and there is also evidence that living donor liver procurement using WJS is secure [15].

Other devices currently used to dissociate the liver parenchyma in LLR include advanced bipolar cautery, LCS, and CUSA. A retrospective comparison of WJS and CUSA revealed that the intraoperative blood loss in WJS was higher while the perioperative blood transfusion rate and other postoperative outcomes were comparable [16]. Otherwise, randomized controlled trials with devices in open liver resection have been reported [17,18] but not in LLR. Except for WJS, each device generates a high fever during liver resection. Thermal damage of the bile duct can occasionally result in bile duct stricture or delayed bile leakage due to ischemia [19]. WJS is superior to other devices in that it does not cause thermal damage.

Concerning LLR in patients with liver cirrhosis, parenchymal resection time was shorter with the advanced bipolar cautery compared with CUSA, but no difference in blood loss or transfusion rate was observed in a propensity score-matching study [20]. In this report, the PM was used in about 90% of instances. The PM is often used to control bleeding during liver parenchymal transection [21]. Although this procedure is clinically proven to be safe, an excessive number of procedures may increase the risk of PHLF in patients with severe liver fibrosis who have inadequate liver function. Anatomical resection of the tumor-bearing portal territory is oncologically appropriate in HCC [22], but non-anatomical resection is often performed in patients with severe liver fibrosis who are concerned about remnant liver function. In anatomical resection, the bleeding is often managed without the PM after the inflow blood vessel is ligated. On the other hand, the PM is performed throughout non-anatomical resections in many cases. Importantly, if hemostasis takes time in patients with liver fibrosis, the quantity of bleeding increases, the resection time is lengthened, and the number of PMs increases, resulting in a vicious cycle. In the current study, the taping of the hepatoduodenal ligament was performed and the PM was planned as necessary, but the bleeding could be managed without the PM. This is thought to be the consequence of preventing vascular injury using WJS and achieving effective hemostasis by SLiC and/or the SLiC-WOC method [10,11].

This study has some limitations. First, the number of cases is small. The second limitation is that it is impossible to determine whether WJS is actually superior to other devices because WJS has not been compared.

## Conclusions

We demonstrated the safety and efficacy of laparoscopic liver resection utilizing WJS in patients with liver fibrosis. Using WJS, the intrahepatic vessels in the fibrotic liver were better exposed and hemostasis was better controlled during laparoscopic liver resection. With this approach, the necessity of Pringle’s maneuver and intraoperative blood transfusion was minimized. This technique could lead to a safer procedure for laparoscopic liver resection even in fibrotic liver conditions.
